# Strain Sensitivity Control of an In-Series Silica and Polymer FBG

**DOI:** 10.3390/s18061884

**Published:** 2018-06-08

**Authors:** Ricardo Oliveira, Lúcia Bilro, Rogério Nogueira

**Affiliations:** 1 Instituto de Telecomunicações, Campus de Santiago, 3810-193 Aveiro, Portugal; lucia.bilro@av.it.pt (L.B.); rnogueira@av.it.pt (R.N.); 2 Instituto de Telecomunicações and I3N/FSCOSD, Institute of Nanostructures, Nanomodelling and Nanofabrication, Physics Department of University of Aveiro, 3810-193 Aveiro, Portugal

**Keywords:** fiber Bragg grating (FBG), polymer optical fiber (POF), optical fiber sensor (OFS)

## Abstract

This work reports on the use of an in-series silica and polymer fiber Bragg grating (FBG) to control the FBG strain sensitivities and enhance in the case of the polymer fiber Bragg grating (PFBG). Due to differences in the Young’s Modulus of the fibers employed, the amount of strain is unequally distributed in each fiber section. By acting on the silica fiber length, it was possible to control the strain sensitivity of the two FBGs, allowing a polymer FBG strain sensitivity much higher than the one found in the elementary fiber to be obtained. The influence of the diameter of the polymer fiber on the strain sensitivities of the FBGs was also investigated. Results have shown that, besides the strain sensitivity control, an even greater improvement in the PFBG strain sensitivity can be achieved.

## 1. Introduction

Fiber Bragg gratings (FBGs) are a well-known fiber optic device that has been attracting significant attention, not only in the research community, but also in industry [1]. The particularities of FBGs have caused them to be widely explored in the sensing area. Indeed, unlike electric sensors, FBGs can offer the following: Immunity to electromagnetic interference; compact size; high sensitivity; simple fabrication; long distances; high resolution; multiplexing capabilities; and the ability to respond to a variety of measurands. Such advantages have led to the use of FBGs in, for instance, the oil industry [2], intrusion detection systems [3], structural health monitoring on aircraft structures, wind turbines and civil engineering structures [4].

Up to now, the majority of FBG sensors that have been reported are based on silica fibers. However, when recorded in polymer optical fibers (POFs), two promising technologies are combined. POFs can offer key advantages over the traditional silica fiber. Among these advantages is their lower Young’s modulus (*E*), which, for the most popular fiber material, polymethylmethacrylate (PMMA), is around 3.2 GPa [5], while silica fibers can reach values of 70 GPa [6]. This property allows POFs to monitor soft materials, a capacity that the stiffer silica fiber does not have. Additionally, a lower *E* value makes POFs appealing for high sensitivity hydrostatic pressure sensors [7]. Other important characteristics, such as their high failure strain, which can reach values above 6% [8], the ability to be humidity sensitive or insensitive [9] and a much higher sensitivity to temperature change, make this fiber optic technology very promising for sensing applications.

One of the most widely used FBG sensors reported on to date is the strain sensor. This type of sensor acts by changing the resonant wavelength when stress is applied to the FBG. This property has been reported to fabricate other types of sensors, such as magnetic field sensors, accelerometers, tilt sensors, liquid level sensors, etc. In all of these sensors, the FBG is attached to a moving part and the strain imposed to the fiber is converted to the units of the external property that is being applied. Some works have attempted to improve [10,11,12,13,14] and control [14,15] FBG strain sensitivity. In the works presented in [10,11,12,13], the sensitivity enhancement is performed by etching the fibers containing the FBGs, which leads to a higher density of stress per unit area and thus an enhancement of FBG strain sensitivity. However, all of them report on the use of fibers with a thin diameter (ranging from 12 to 60 µm), which leads to issues concerning either fiber manipulation in practical applications or the provision of a low dynamic range, which is due to the fragility of the structures, especially in those employing silica fibers. The works related to strain sensitivity control report on the use of a fiber taper [15] and a metal stick [14] with an in-series FBG to create an unequal strain distribution on each part of the total gauge length. In the first work, the unequal strain distributions impose a higher density of stresses on the fiber taper than on the raw FBG. Unfortunately, the maximum FBG strain sensitivity is limited to that found in standard FBGs. In the second work, it is reported that a metal stick allows for the control of the thermal expansion of the sensing head and also the improvement of the density of stresses on FBGs, promoting a higher strain sensitivity. However, such a scheme imposes a huge amount of stress on the silica FBG, which, considering their low failure strain (<1% [6]), leads to the sensor offering a low measurement range.

In this work, we report on the use of in-series silica and polymer fibers for the control of FBG strain sensitivity. To achieve this, we will demonstrate that the unequal strain distributions in each fiber section of the total gauge length is the function of *E*, the cross-sectional area and the length of the fibers employed. These properties will reveal the possibility of controlling FBG strain sensitivities and also increasing the FBG inscribed in the POF.

## 2. Sensor Fabrication

**Figure 1:**
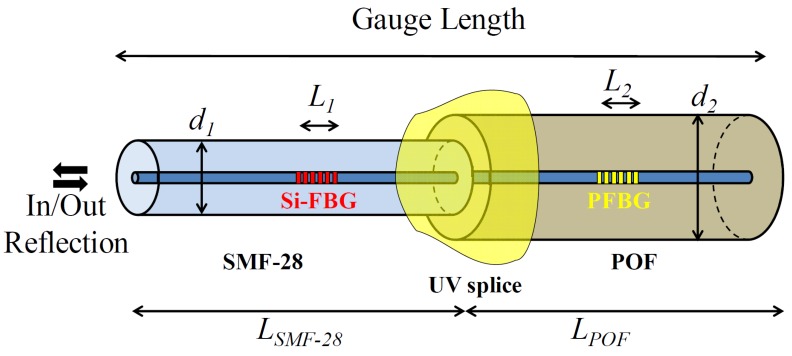
Schematic of the sensor, employing an FBG in a POF, in-series with a silica FBG by means of a UVsplice using a photopolymerizable adhesive. *L_i_*, and *d_i_* represent the length and diameter of the fibers, respectively.

The proposed fiber sensor head can be seen in Figure 1. It is composed of a microstructured polymer optical fiber (mPOF), spliced to a silica fiber through a photopolymerizable resin.

The fiber sensor was constructed by employing a combination of two FBGs, one in a standard silica single mode fiber (SMF-28) and another in an mPOF. The SMF-28 fiber is composed of an 8.2 µm core and a 125 µm cladding, and supports a single mode behavior in the 1550 nm region. The mPOF, made from undoped PMMA, was bought from Kiriama Pty Ltd. (Sydney, Australia). The fiber is composed of a 247 µm cladding, 18 µm core and six air hole layers in a hexagonal arrangement that runs along the length of the fiber. The air holes have diameters of 3.2 µm and are spaced by a pitch of 6.2 µm, giving a total of 162 holes. The fiber supports a few-mode behavior at the C-band and the attenuation in this region is estimated to be around 3 dB/cm.

The Bragg gratings in the silica and mPOF were inscribed through the phase mask method, employing a Krypton Fluoride UV laser, with a repetition rate of 1 and 500 Hz and an exposure time of 10 and 20 s, with phase masks of 1061.56 nm and 1073.66 nm, respectively. Further descriptions of the inscription process of the polymer fiber Bragg grating (PFBG) can be found in [16]. The length of the grating was imposed by the slit aperture, which was set to 4.5 mm. Additionally, in order to avoid residual stresses, left during the drawing process of the mPOF, and to improve the PFBG strain sensitivity [17], the fiber was annealed at 70 °C for 12 h prior to, and after, the inscription process.

In order to assemble the sensor for the strain tests, the silica fiber, which acted as a light delivering system, was cleaved at 1 cm from the FBG with an 8° angle, for the sake of reducing back reflections. A length of 1.2 m of the fiber was also uncoated in the strain characterization. The mPOF was cleaved at 1 cm from the PFBG and prepared following the descriptions found in [18]. The silica and POF terminals were then aligned through their centers, and a drop of photopolymerizable resin (NOA86H, from Norland Products, Inc., Cranbury, NJ, USA) was placed between the fibers. The gap between the fibers was adjusted to its minimum in order to avoid the formation of Fabry Perot cavities that could deteriorate the reflection signal of both the silica and polymer FBGs. To set the resin, a handheld UV source (Opticure LED 200, from Norland Products, Inc.), with a wavelength of 365 nm and a fluence of 2.5 W/cm^2^, was used to illuminate the region for 10 s. It is worth noting that the resin employed was chosen due to its optimum adhesion to polymer and silica materials, offering a Young’s modulus of ~2.5 GPa [19], which is close to the value found in PMMA fibers [5]. One feature of the fiber connection employed in this work is related to the induced coupling loss between the silica fiber and the mPOF. However, from our previous work [16], it can be observed that the grating strength measured in reflection through this kind of connection, is about 20 dB. Therefore, the coupling losses between the fibers will not be an issue for the sensing scheme employed in this work (measuring the peak Bragg wavelength shift).

## 3. Sensor Characterization

The strain characterization tests were performed in two stages, one considering the sensing head composed of raw fibers and another considering the mPOF with a reduced diameter.

The strain test was performed on the sensing head composed of raw fibers by gluing the raw mPOF containing the PFBG to a static stage and the FBG silica fiber to a linear stage (MFA-CC from Newport Corporation, Irvine, CA, USA) consisting of a 1 µm resolution. The length of the mPOF was fixed at 2.7 cm, while the silica fiber length was changing, in order to achieve the total gauge lengths of: 1.00; 0.80; 0.60; 0.50; 0.40; 0.30; 0.20; 0.10 and 0.05 m. The characterization was made by straining the sensing head in steps of 200 µε, up to a value of 1200 µε, considering room temperature and humidity conditions.

In order to verify the influence of the cross-sectional area of the mPOF on the strain sensitivity performance of both FBGs, an etching process on the entire length of the mPOF was employed. To achieve this, acetone was employed for long enough to etch the cladding region, without reaching the microstructure. The time taken was 15 min, and the diameter was measured to be approximately 130 µm. The strain characterization tests were then repeated for the same gauge lengths, considering strain steps of 50 µε, up to a value of 400 µε, and room temperature and humidity conditions.

The signal was measured in reflection through the silica fiber that was connected to an interrogator system (sm125-500, from Micron Optics, Atlanta, GA, USA), with 1 pm resolution.

## 4. Results

**Figure 2:**
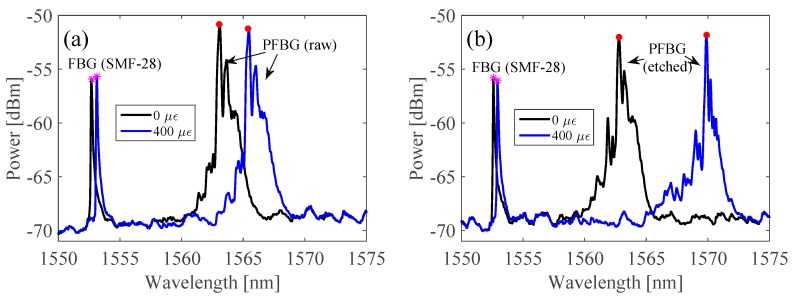
Reflection spectra obtained, when the fibers were unstrained and strained with 400 µε, for the sensing head with: (**a**) raw and (**b**) etched POF, considering 2.7 cm mPOF and 1.00 m gauge lengths.

Results concerning the reflection spectra of the unstrained and strained fibers under 400 µε conditions, considering a total gauge length of 1.00 m, for the raw and etched mPOF sensing heads, can be seen in Figure 2a,b, respectively.

**Figure 3:**
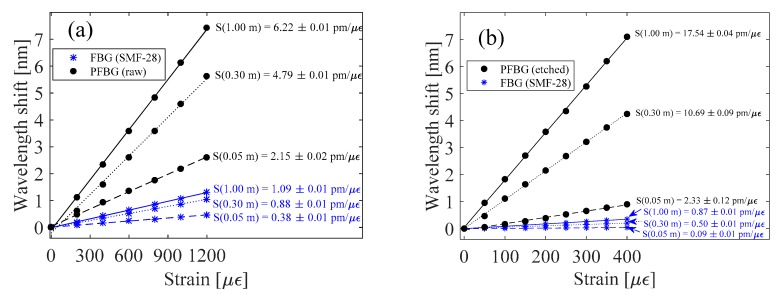
Sensitivity results of the silica and polymer FBGs, considering 2.7 cm mPOF and gauge lengths of 0.05, 0.30 and 1.00 m, for the sensing head with: (**a**) raw and (**b**) etched mPOF.

From Figure 2a,b, it can be seen that both the silica and polymer FBGs follow a red-shift as the strain increases, which is the peak Bragg wavelength shift (the pink stars and red dots representing the silica and polymer FBGs, respectively), more noticeable in the case of the mPOF. The result is a consequence of the difference in length, cross-sectional area and Young’s modulus of the fibers involved. In fact, silica fibers have a Young’s modulus of about 70 GPa [6], while PMMA-based POFs are commonly found to have 3.2 GPa [5]. This difference results in unequal strain distributions on each fiber section, which is higher in PMMA fibers and lower in silica ones. Additionally, it can be seen that, with the same strain condition, the PFBG spectrum of the sensing head composed of the etched mPOF has a larger Bragg wavelength shift than the raw mPOF sensing head. This is due to the reduced cross-sectional area of the etched mPOF sensing head. Thus, the strain on the mPOF is higher than that on the silica fiber, which accounts for the higher and lower Bragg wavelength shifts of the polymer and silica FBGs, respectively. The aforementioned results concerning the resonant Bragg wavelength shift response of the strain tests with different gauge lengths, considering the sensing head with the raw and etched mPOF, can be better visualized from Figure 3a,b.

Linear fits, adjusted to the experimental Bragg wavelength shifts with three gauge lengths (i.e., 1.00; 0.30; and 0.05 m), for the raw and etched mPOF sensing heads are presented in Figure 3a,b, respectively. From Figure 3a,b, it can be seen that the sensitivity of both FBGs increased as the total gauge length increased (i.e., 0.38 to 1.09 for the silica FBG and 2.15 to 6.22 pm/µε for the PFBG, considering 0.05 and 1.00 m gauge lengths). However, if one compares the sensitivity of the FBGs found in each fiber sensing element, one can observe that the silica FBG strain sensitivity always remains below the standard value (measured as 1.18 pm/µε), while the PFBG strain sensitivity is 5 times greater compared with the standard PFBG raw value (measured as 1.21 pm/µε). If one reduces the diameter of the mPOF from 247 to 130 µm (~0.5 times), the PFBG strain sensitivity, considering the gauge length of the mPOF, reaches a value of 1.27 pm/µε. This improvement is in agreement with the results demonstrated in [13]; however, differences may appear due to the dissimilarities of the fibers. On the one hand, if one considers the gauge length of the etched fiber combined with the silica fiber, the PFBG strain sensitivity can be increased in a higher proportion. This is demonstrated in Figure 3b, where it can be seen that the PFBG strain sensitivity reaches values ranging from 2.33 to 17.54 pm/µε with gauge lengths ranging from 0.05 to 1.00 m, respectively. Additionally, it can be seen that, for each fiber length, the PFBG strain sensitivity is 3 times greater when compared with that found in the raw POF sensing device. It is worth noting that by further reducing the diameter of the mPOF, one can easily improve the PFBG strain sensitivity to attain even higher values. However, in the presented work, further etching the PFBG would damage the fiber microstructure as well as the light guiding properties. On the other hand, considering the silica FBG strain sensitivity, one can see that the opposite of what happens to the PFBG occurs, i.e., a decrease in sensitivity with small POF diameters.

**Figure 4:**
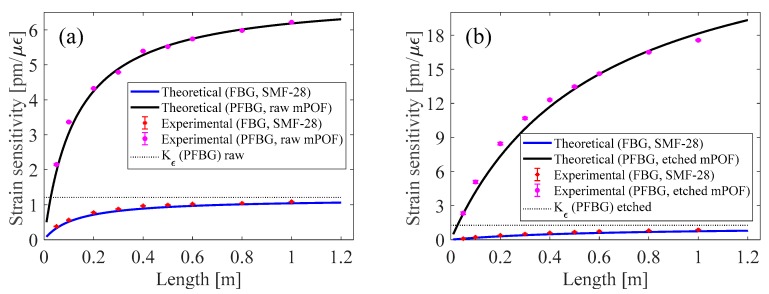
Theoretical and experimental results obtained from Equations (2) and (3) with a 2.7 cm mPOF, considering different gauge lengths, for the sensing head composed of: (**a**) raw and (**b**) etched mPOF.

Results concerning the experimental strain sensitivity values of the silica and polymer FBGs, taking into account different gauge lengths (i.e.,: 1.00; 0.80; 0.60; 0.50; 0.40; 0.30; 0.20; 0.10 and 0.05 m) and considering the sensing head composed of raw and etched fibers, are represented by the marker points in Figure 4a,b, respectively.

In order to theoretically analyze the strain sensitivity evolution of the two FBGs under investigation, and corroborate them with the experimental results, similar deductions as those presented for an in-series silica FBG with a tapered fiber, found in [15], were followed. To achieve this, it was necessary to consider that the stress along the gauge length is unequally distributed on each fiber sensing element and depends on the cross-sectional areas (*A*_i_) and Young’s modulus (*E*_i_) of the fibers involved, which can be expressed as:

(1) $$\varepsilon_{1}E_{1}A_{1}=\varepsilon_{2}E_{2}A_{2},$$

where *ε*_i_ refers to the strain, and subscript 1 and 2 refer to the silica and polymer FBG, respectively. Taking into account the longitudinal strain on each part of the sensing head, and considering the strain characterization at constant humidity and temperature conditions, one can theoretically estimate the strain sensitivity of the silica FBG as:

(2) $$K_{\varepsilon ,1}=K_{\varepsilon }(SiFBG)\frac{L_{1}+L_{2}+L_{SMF-28}+L_{POF}}{(L_{2}+L_{POF})\frac{E_{1}A_{1}}{E_{2}A_{2}}+L_{1}+L_{SMF-28}},$$

and the PFBG as:

(3) $$K_{\varepsilon ,2}=K_{\varepsilon }(PFBG)\frac{L_{1}+L_{2}+L_{SMF-28}+L_{POF}}{(L_{1}+L_{SMF-28})\frac{E_{2}A_{2}}{E_{1}A_{1}}+L_{2}+L_{POF}},$$

where *K_ε_*(*SiFBG*) and *K_ε_*(*PFBG*) refer to the elementary strain sensitivity of the silica and polymer FBGs, respectively. *L*_1_, *L*_2_, *L_SMF-_*_28_ and *L_POF_* define the length of the silica FBG, PFBG, silica fiber, and POF, respectively, and their sum represents the total gauge length.

In order to plot the functions presented in Equations (2) and (3), the strain sensitivity values found in each elementary fiber were used: *K_ε_*(*SiFBG*) = 1.18 pm/µε, *K_ε_*(*PFBG* raw POF) = 1.21 pm/µε and *K_ε_*(*PFBG* etched POF) = 1.27 pm/µε. Additionally, the cross-sectional area of the mPOF was calculated, considering the clad area without the 162 air holes. Results showing the theoretical strain sensitivity of the silica and polymer FBGs, considering the sensing head composed of the raw and etched fibers, can be seen in Figure 4a,b, respectively.

As can be seen from Figure 4a,b, the theoretical strain sensitivity of the silica and POF fibers matches the ones found experimentally. Additionally, it can be seen that the strain sensitivity limit reaches its maximum values at lengths greater than 1.00 m. By analyzing the limit of Equations (2) and (3), when *L_SMF-_*_28_ tends to be infinite, the silica FBG reaches its elementary strain *K_ε_SiFBG*), while the PFBG is given by *K_ε_*(*PFBG*)*E*_1_*A*_1_/*E*_2_*A*_2_, which, with respect to the current fibers employed, gives values of 6.98 and 28.67 pm/µε for the raw and etched mPOF sensing heads, respectively.

Considering the sensing head composed of the etched POF, the strain sensitivity for a total gauge length of 20 cm is higher than that achieved for the theoretical maximum gauge length of the raw mPOF sensing head. This demonstrates the influence of the diameter of the mPOF on the strain sensitivity of a sensing head composed of two dissimilar fibers.

We stress that the fiber sensors reported in this work were intended to show the proof of a concept of the strain sensitivity control of silica and polymer FBGs, and also its enhancement in the case of PFBG. Nonetheless, due the deployment and availability of PMMA-based POFs, we employed this kind of POF in our work. However, PMMA can absorb the water from the environment, which poses cross sensitivity issues. Additionally, both silica and polymer FBGs are sensitive to temperature, adding another parameter to the system. To solve issues related to the water absorption of the PMMA, a new class of humidity-insensitive polymer fiber materials, such as the ones demonstrated in [20,21], could be employed. Regarding cross sensitivity to temperature, since the proposed sensors are formed by combining two in-line FBGs with different sensitivities, a 2 × 2 matrix could be employed to simultaneously measure strain and temperature. Nevertheless, other methods to discriminate the aforementioned parameters could be employed. An example of that can be given by adding another silica FBG before and after the strain setup. Thus, the first silica fiber measures only temperature, the second measures temperature and strain, and the third (PMMA PFBG) measures temperature, strain and humidity. By combining all these sensitivities in a 3 × 3 matrix, all three parameters could be discriminated.

Regarding applications, this fiber sensing device could be employed in situations where strain is applied between two points (crack monitoring), and where the strain sensitivity needs to be higher than that found in single fiber elements. Furthermore, it is worth noting that the proposed sensor needs to move freely, since any contact with external surfaces in-between the fiber could modify the strain measurements.

For practical applications, the strain range of the developed sensing heads is crucial. In this work, the strain sensitivity increase in the PFBG is related to the strain on the mPOF, which is higher than that on the silica fiber. This occurs due essentially to the Young’s Modulus and length of the POF, which are lower than those of the silica fiber. Thus, the strain range of the sensor is a function of the POF limits. Considering the sensor operating in the elastic regime, and knowing that POFs have an elastic limit of up to 20,000 µε [22], one can therefore estimate the total strain range of the sensor using the elementary longitudinal strain that appears in Equation (3):

(4) $$\varepsilon_{tot}=\frac{L_{2}+L_{POF}+(L_{1}+L_{SMF-28})\frac{E_{2}A_{2}}{E_{1}A_{1}}}{L_{1}+L_{2}+L_{SMF-28}+L_{POF}}\varepsilon_{2},$$

**Figure 5:**
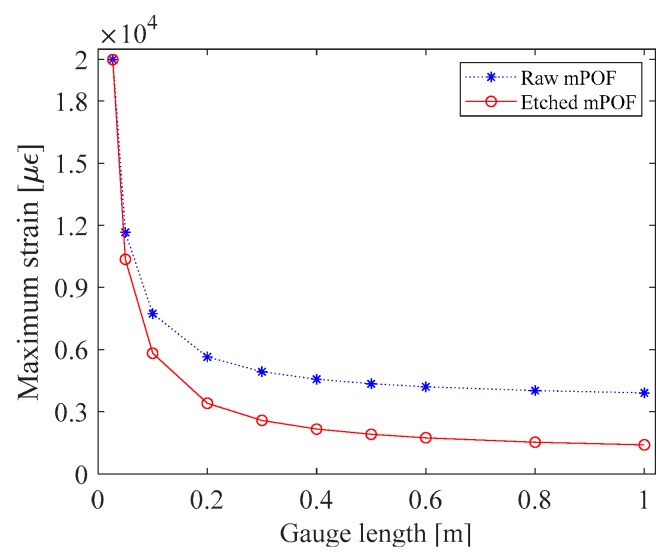
Maximum strain on the fiber sensing heads, considering an elastic regime.

By estimating the total strain range in this way, and considering the fiber lengths employed in this work, the strain limit of the fiber sensor composed of the raw and etched mPOFs can be determined, as represented in Figure 5 by the blue and red curves, respectively.

As can be observed in Figure 5, the strain limit, considering the operation of the sensing head in the elastic regime, decreases as the gauge length increases. This behavior was expected, since one of the goals of this work was to show that POFs are subjected to a higher density of strain in the presence of an increasing silica fiber length, which leads to a higher PFBG sensitivity. Therefore, a higher density of strain on an mPOF leads to have a lower strain range. It can also be seen in Figure 5 that the maximum strain on the sensing head composed of the raw mPOF is higher than that on the etched mPOF, since the maximum strain is proportional to the cross-sectional area of the POF, as described in Equation (4). However, the best sensitivity is achieved in the thin mPOF sensing head. In sum, whenever a sensor of this type is used, it is necessary to know the limits within which the sensor operates in order to design a sensing head with the best sensitivity.

## 5. Conclusions

This work reports on the strain sensitivity control of FBGs in polymer and silica fibers by employing them in a dual in-line scheme. Parameters, such as the Young’s modulus of the fibers, diameter of the POF and length of the silica fiber, were taken into account. From this, we have shown that the sensitivity of silica and polymer FBGs can be controlled by adjusting the silica fiber length. Since the Young’s modulus of the silica fiber was larger than that of the POF, the strain distribution was more pronounced on the POF than on the silica fiber. Consequently, the PFBG strain sensitivity achieved values much higher than those found in elementary PFBG. Nevertheless, the influence of the diameter of the POF was also investigated, showing that, besides controlling the FBG strain sensitivity, an improvement in the PFBG was also observed, paving the way for high sensitivity PFBG strain sensors.
